# Displacement detection is suppressed by the post-saccadic stimulus

**DOI:** 10.1038/s41598-020-66216-1

**Published:** 2020-06-09

**Authors:** Shuhei Takano, Kazumichi Matsumiya, Chia-huei Tseng, Ichiro Kuriki, Heiner Deubel, Satoshi Shioiri

**Affiliations:** 1 Graduate School of Information Sciences, Tohoku University, 6-3-09 Aramaki aza Aoba, Aoba-ku Sendai, 980-8579 Japan; 2 Research Institute of Electrical Communication, Tohoku University, 2-1-1 Katahira, Aoba-ku, Sendai 980-8577 Japan; 3 Department Psychologie, Ludwig-Maximilians-Universität, Leopoldstr, 13 D-80802 München Germany

**Keywords:** Human behaviour, Motion detection, Saccades, Oculomotor system, Visual system

## Abstract

To establish a perceptually stable world despite the large retinal shifts caused by saccadic eye movements, the visual system reduces its sensitivity to the displacement of visual stimuli during saccades (e.g. saccadic suppression of displacement, SSD). Previous studies have demonstrated that inserting a temporal blank right after a saccade improves displacement detection performance. This ‘blanking effect’ suggests that visual information right after the saccade may play an important role in SSD. To understand the mechanisms underlying SSD, we here compare the effect of pre- and post-saccadic stimulus contrast on displacement detection during a saccade with and without inserting a blank. Our results show that observers’ sensitivity to detect visual displacement was reduced by increasing post-saccadic stimulus contrast, but a blank relieves the impairment. We successfully explain the results with a model proposing that parvo-pathway signals suppress the magno-pathway processes responsible for detecting displacements across saccades. Our results suggest that the suppression of the magno-pathway by parvo-pathway signals immediately after a saccade causes SSD, which helps to achieve the perceptual stability of the visual world across saccades.

## Introduction

Our retinas are grossly inhomogeneous with a small central visual field with high accuracy and a large peripheral field with low spatial resolution. To efficiently collect information from the surroundings, saccadic eye movement shift gaze locations several times per second. This is a crucial function that enables us to select information at a number of locations in the external world. However, this strategy faces potentially serious problems. First, the retinal image becomes blurred during a saccade and this could impair visual perception. Second, across the saccade, the object positions shift on the retina, causing a large change in the retinal image which leads to the challenge to integrate the images before and after the saccade. How the visual system achieves the perception of a stable and consistent visual world across a saccade is one of the essential questions in visual science.

It is known that visual sensitivity in a variety of tasks decreases during saccades including light flash detection^1,2^, displacement^3^, motion^4^, color change^5^, and so on. This phenomenon is called saccadic suppression, or saccadic omission by forward and/or backward masking^6,7^. Saccadic suppression may prevent the perception of a blurred retinal image caused by the saccade^8,9^. The second problem, which is related to the shifting of the retinal projections of objects is more difficult to solve, thus presenting the challenge of integrating retinal images before and after the saccade. Obviously, the removal of retinal information during saccades cannot stabilize the visual world because the different retinal images before and after saccades are highly visible. Therefore, a mechanism is hypothesized to estimate the saccade-induced shift in a retinal image from an efference copy of the motor command of the eye movement to compensate for image shifts. After a readjustment of the stimulus location in space, the mechanism is able to integrate retinal images before and after saccades. However, since the actual saccade amplitude and the saccade amplitude predicted from an efference copy are not always the same^10^, an object could be perceived as displaced during a saccade even without physical displacement if the visual system fully trusts the efference copy. As a possible solution to this problem, it was discovered that the visual system does not detect a small displacement of an object during a saccade even if it was easily detected during fixation^3^, a phenomenon called saccadic suppression of displacement (SSD). Having a certain amount of tolerance for target displacements across saccades could be a simple way to achieve effective perceived stability. Indeed, people who have larger distributions of saccade landing points (that is, potentially larger errors to rearrange retinal inputs in the spatial coordinates after saccades) have stronger SSD^12,13^. Thus, SSD could play an important role with regard to visual stability across saccades.

The blanking effect^14^ was an important milestone for our understanding of visual stability. The blanking effect is a phenomenon in which our detection performance of a target displacement during a saccade is improved by inserting a temporal blank (50–300 ms) during the saccade and before the target reappears at a new position. This phenomenon suggests that signals about target displacements are indeed available after the saccade, but cannot be used for displacement detection when the saccade target is present at the end of the saccade, i.e., without a post-saccadic blank. The displacement can be perceived however when the suppression is relieved by inserting a post-saccadic blank. The blanking effect supports the assumption that SSD is an active process to avoid any erroneous displacement perception caused by inaccurate saccades and/or inaccurate efference copies of the saccade commands, because it demonstrates that there is sufficient information to detect the displacement after the saccades.

Previous studies have shown that the magnocellular pathway (M-pathway), which responds to stimulus motion, is selectively suppressed during saccades^11,15^ and that low-level motion is suppressed across saccades^4^. Based on these findings, we assume in our model that M-pathway signals are suppressed around saccades. The blanking effect indicates that the presence of a visual stimulus after the saccade is critical for SSD. Blanking or removing the target immediately after the saccade appears to inhibit the activity of the mechanism that suppresses the magnocellular pathway during a saccade. Interestingly, a recent study on the influence of target luminance contrast on displacement detection during a saccade showed that the contrast dependence of displacement detection is different for blank and no-blank conditions: displacement detection is improved by an increase in target contrast both with and without a blank, but the effect is stronger with a blank^16^. This study also demonstrated that there was no blanking effect with very low stimulus contrast, and for isoluminant stimuli. The difference in contrast dependence between the blank and no-blank condition seems to indicate that two different mechanisms contribute to detection and the suppression of detection, and that the suppression mechanism is not effective with a low contrast stimulus. If the magnocellular pathway is the one to detect displacement or motion, and if it is suppressed during saccades, one possible source of SSD may be the parvocellular pathway (P-pathway). Indeed, it is known that the M-pathway has higher contrast sensitivity than the P-pathway while the M-pathway output saturates with a lower contrast than that of the P-pathway.

To investigate how stimulus strength influences displacement detection, we measured the effect of pre- and post-saccadic target contrast on displacement detection across saccades. Because the presentation timing and position of the pre-saccadic target was the same under both the blank and no-blank conditions, any difference in detection must be due to the post-saccadic target. In order to investigate the specific contributions of pre- and post-saccadic stimulus strength separately, we measured displacement detection with various combinations of pre- and post-saccadic target contrasts. We found that displacement detection was better with a higher-contrast pre-saccadic target, which was in line with the general expectation that higher signal amplitudes should lead to better signal/noise ratios. Surprisingly, but consistent with the possible suppression by P-pathway, we found that increasing the post-saccadic target contrast impaired the performance under the no-blank condition, while there was no clear effect of contrast under the blank condition. This suggests that a stronger signal from a post-saccadic target suppresses the mechanism used to detect displacement.

## Results

### Influence of contrast on displacement detection

Observers were instructed to make a 17.8° rightward saccade from a fixation point to the target. The target was extinguished with saccade onset and presented at a position 0.33° to the left or right of target original position, either immediately (no-blank condition) or following a 100 ms delay (blank condition). Observers reported whether the target was displaced to the left or the right. The contrast value of the pre- and post-saccadic targets were varied in each trial. Because the contrast sensitivity differs for foveal and peripheral vision, the stimulus contrast of pre- and post-saccadic targets was normalized to the detection threshold of individual observer at each retinal location (see Supplementary Fig. S1 online).

**Figure 1:**
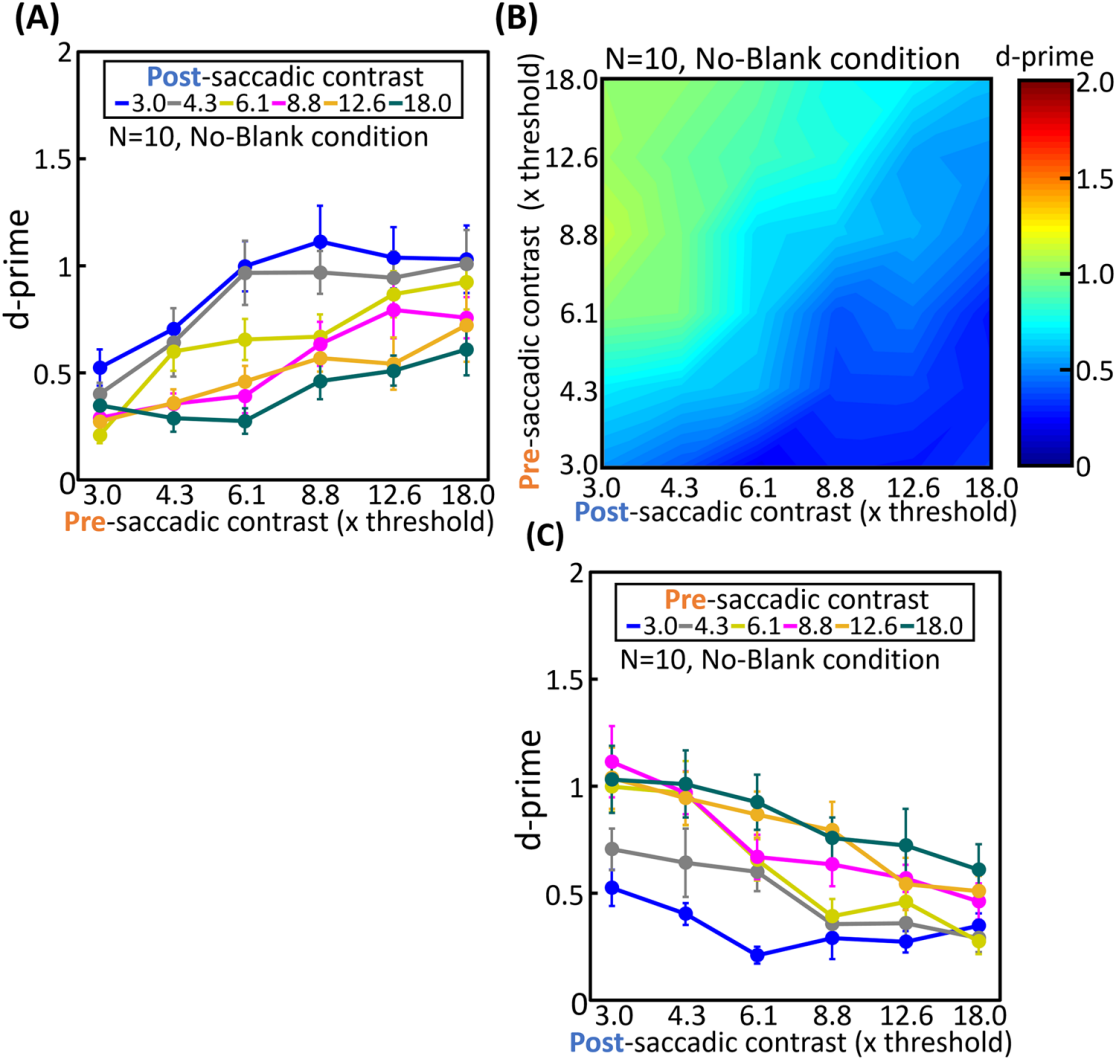
Influence of pre- and post-saccadic target contrast on displacement detection sensitivity under no-blank condition. Observers reported the direction of a target displacement across a saccade (left or right). Pre- and post-saccadic target contrasts were varied independently. d’ was calculated for each contrast condition as an indicator of the displacement detection sensitivity. (**A**) Average d’ as a function of pre-saccadic contrast. Each curve corresponds to different post-saccadic contrast conditions. The error bars represent S.E.M. (**B**) Heat map of average d’ in each contrast condition. The vertical axis indicates pre-saccadic contrast and the horizontal axis indicates post-saccadic contrast. (**C**) Average d’ as a function of post-saccadic contrast. Each curve corresponds to different pre-saccadic contrast conditions. The error bars represent S.E.M.

**Figure 2:**
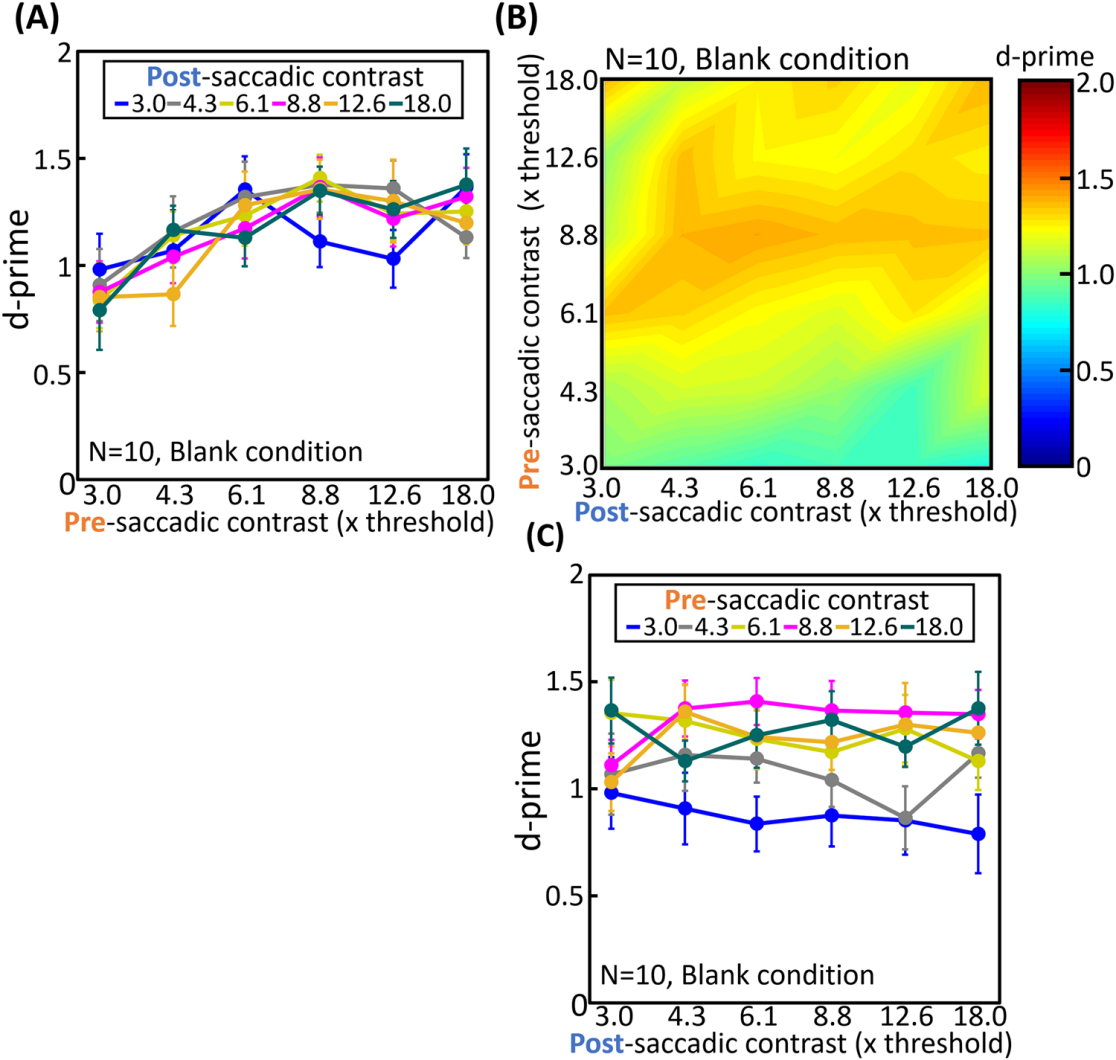
Influence of pre- and post-saccadic target contrast on displacement detection sensitivity under blank condition. d’ was calculated for each contrast condition as an indicator of the displacement detection sensitivity. (**A**) Average d’ as a function of pre-saccadic contrast. Each curve corresponds to different post-saccadic contrast conditions. The error bars represent S.E.M. (**B**) Heat map of average d’ in each contrast condition. The vertical axis indicates pre-saccadic contrast and the horizontal axis indicates post-saccadic contrast. (**C**) Average d’ as a function of post-saccadic contrast. Each curve corresponds to different pre-saccadic contrast conditions. The error bars represent S.E.M.

The detection sensitivity for target displacements across a saccade was expressed by d’ in each contrast condition. We defined a correct report for the right (or left) displacement of the target as a hit, and an incorrect report for the left (or right) displacement of the target as a false alarm. d’ for the no-blank and blank conditions are shown in Figs. 1 and 2, respectively. To analyze the influences of pre- and post-saccadic contrast, 2-way ANOVA (pre/post and contrast level) was used in the no-blank and blank condition respectively. In the no-blank condition, the detection sensitivity decreased significantly with the post-saccadic contrast (F(5,45) = 16.23, p < 0.001, Fig. 1C), while it improved with the pre-saccadic contrast (F(5,45) = 18.54, p < 0.001, Fig. 1A). In the blank condition, the detection sensitivity improved with the pre-saccadic contrast (F(5,45) = 12.45, p < 0.001, Fig. 2A) but not affected by the post-saccadic contrast (F(5,45) = 0.26, p = 0.93, Fig. 2C).

The improvement in detection sensitivity with the pre-saccadic contrast found for both the blank and no-blank conditions agrees with the typical contrast effect on visual functions, namely, performance is better with a stronger stimulus. On the other hand, increasing the contrast of the post-saccadic target impaired the performance in the no-blank condition, and there was no effect of post-saccadic contrast in the blank condition. This implies that a post-saccadic stimulus strengthens the SSD. In the following section, we consider the underlying mechanism of SSD based on these findings.

### Modeling contrast effects on SSD

Retinal information is transferred to the visual cortex through two major pathways that are functionally distinct: the magnocellular pathway (M-pathway) and the parvocellular pathway (P-pathway). Psychophysical studies have suggested that the M-pathway is significantly suppressed around saccades^11,15^. Our results show that luminance information from an object presented immediately after saccades strongly affects displacement detection performance, as if the suppression for the M-pathway was strengthened by increasing the amount of the luminance contrast. If the M-pathway is suppressed around the saccades, the P-pathway should be dominant right after the saccades, that is, the post-saccadic retinal information is processed mainly through the P-pathway. The sensitivity reduction with an increase in the post-saccadic contrast suggests that the P-pathway activity suppresses displacement detection (that is, there is stronger suppression with a larger signal in the P-pathway). To examine this hypothesis, we built a model based on the assumption that the P-pathway is dominant immediately after a saccade. If we assume that there are only two visual pathways from retina to cortex, ignoring other pathways such as extrastriate pathways, one of the simplest models is that the M-pathway signals are suppressed by the signals from the P-pathway or higher level visual areas that receive input from the P-pathway signal. We fit the model to the current results and evaluate parameters related to the characteristics of the M-pathway and P-pathway. The estimated parameters related to the contrast characteristics of the M-pathway and P-pathway are consistent with the results of a physiological study^17^.

The response of each pathway to target contrast was expressed using functions that have been used to simulate the contrast response of M-type and P-type LGN cells^17,18^, and also used to compare with psychophysical results^19^. To estimate the output signal for displacement detection, we used the Reichardt correlation motion detector model^20^, with which we calculated the correlation between the pre- and post-saccadic signals that are mediated by M-pathway based on our hypothesis. While the Reichardt model deals with spatiotemporal stimulation as a motion detector model, we here simply calculate correlation (multiplication) of the contrast responses of M-type cells to the pre- and post-saccadic target, assuming a detector that has appropriate spatiotemporal tuning for the present stimulus. The purpose of the calculation was to estimate the amount of output (in terms of signal amplitude d’) that contributes to displacement detection under each contrast condition. The suppression of displacement detection was represented by dividing the output signal of the detection system by the response of the P-pathway to the post-saccadic target. That is, the larger responses of the P-pathway, the larger denominator of the model and overall model output become lower.

The following equation calculates the output of the model used to express the influence of the pre- and post-saccadic contrast on detection sensitivity.

$${d}^{\text{'}}=\frac{R\times \left(\frac{{k}_{{\rm{pre}}}^{{\beta }_{{\rm{m}}}}}{{k}_{{\rm{pre}}}^{{\beta }_{{\rm{m}}}}+{\alpha }_{{\rm{m}}}^{{\beta }_{{\rm{m}}}}}\right)\times \left(\frac{{k}_{{\rm{post}}}^{{\beta }_{{\rm{m}}}}}{{k}_{{\rm{post}}}^{{\beta }_{{\rm{m}}}}+{\alpha }_{{\rm{m}}}^{{\beta }_{{\rm{m}}}}}\right)}{{S}_{\begin{array}{c}no-blank\\ orblank\end{array}}\times \left(\frac{{k}_{{\rm{post}}}^{{\beta }_{{\rm{p}}}}}{{k}_{{\rm{post}}}^{{\beta }_{{\rm{m}}}}+{\alpha }_{{\rm{p}}}^{{\beta }_{{\rm{p}}}}}\right)+{n}_{0}}$$

where k_pre_ represents the normalized contrast of a pre-saccadic target to individual contrast detection threshold, and k_post_ represents the normalized contrast of a post-saccadic target to individual contrast detection threshold. Parameters α and β represent the half-saturation contrast and the steepness of the contrast dependence, and R is the coefficient used to convert the response to the sensitivity of the displacement detection. The parameter n_0_ is a constant of the non-linear function modeling neural contrast responses, which controls the noise level. The subscripts m and p represent the M-pathway and P pathway, respectively. S represents the contribution of the P-pathway response, which affects the displacement detection. S is assumed to be different for the blank and no-blank conditions following the sensitivity changes (SSD difference) that depend on the presence or absence of a blank (S_No-blank_ > S_Blank_). S_Blank_ was used to examine the relationship between saccade accuracy and suppression effect for each observer.

**Figure 3:**
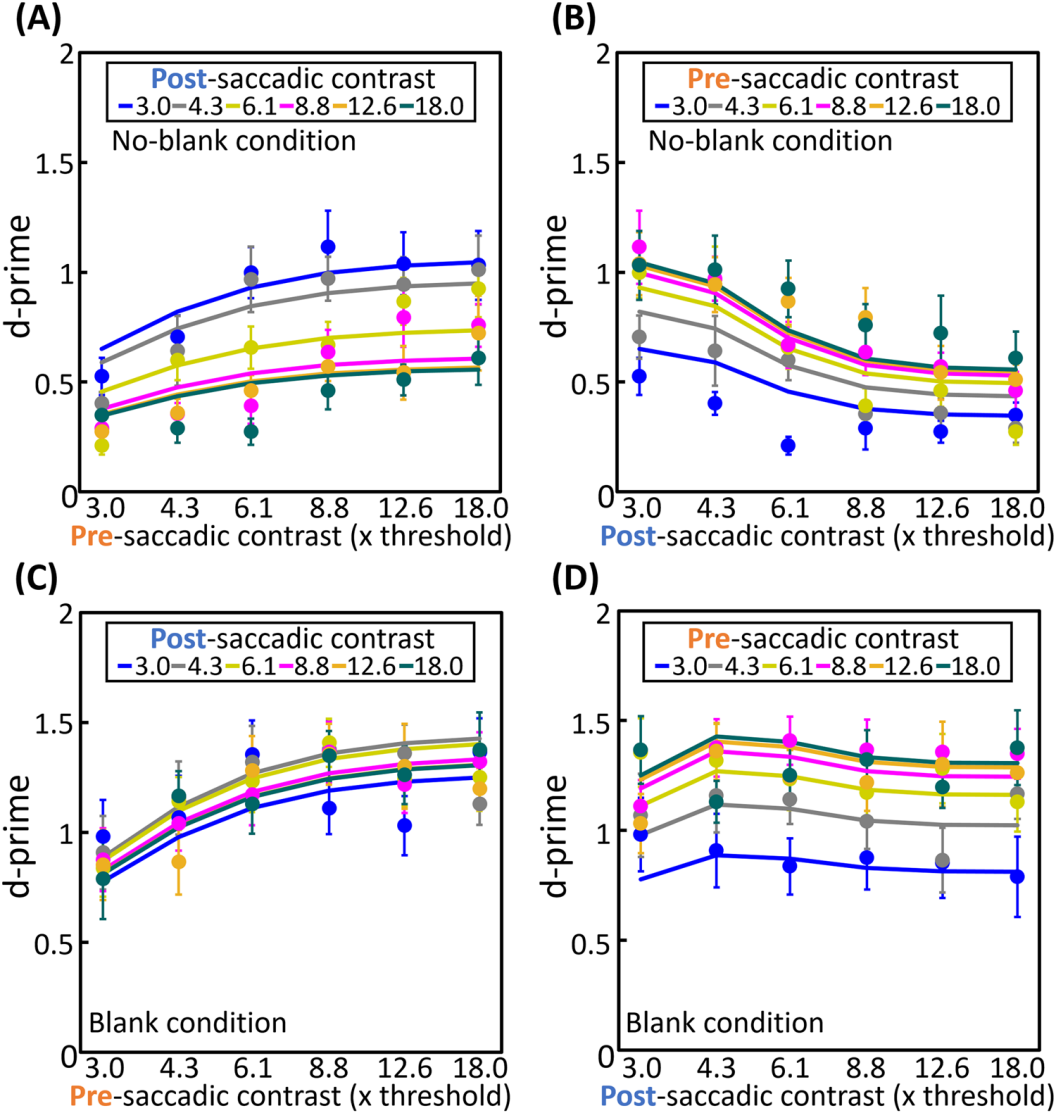
Fitting results of our model. Results of model fitting. The plots show the average d’ over observers and error bars represents S.E.M., which are calculated from actual responses. Each line shows a fitting result. (**A**), (**B**) The results for the no-blank condition. (**C**,**D**) The results for the blank condition.

We fit this model to the data for all conditions averaged over observers by the least-squares procedure with free parameters of k_pre_, k_post_, α_m_, β_m_, α_p_, β_p_, R, S, and n_0_ (Fig. 3, the obtained parameters are shown in Table 1). The goodness of fit was evaluated with a χ^2^ test and the test showed that this model was not rejected with a rejection rate of 5% (χ^2^ (71) = 1.2, p=1.00). According to Table 1, the P-pathway function has a lower contrast response and a smaller half-saturation contrast than the M-pathway function, which corresponds to the difference in α (α_p_ is 2.4 times larger than α_m_). This is consistent with the difference between the contrast response characteristics of P-type and M-type LGN cells reported by a previous study^17^: the half-saturation contrast of P-type LGN cells is approximately 5 times larger than that of M-type LGN cells.

**Table 1:** Fitting parameters of our model.

α_m_	β_m_	R	α_p_	β_p_	S_Blank_	S_No-blank_	n_0_
2.42	2.11	1.84	5.78	3.49	0.54	2.42	0.84

Numbers show the parameters obtained by fitting the proposed model to displacement detection sensitivity data averaged over all observers.

Fitting results indicate that the model provides a good approximation of the influence of pre- and post-saccadic contrast on detection sensitivity. This supports our hypothesis that one of the two pathways contributes to the suppression of displacement detection across saccades while the other contributes to detection. Specifically, the estimated parameters for the two pathways agree with the hypothesis that the P-pathway contributes positively to the suppression around a saccade and the M-pathway contributes to detection.

## Discussion

The current study suggests that it is the retinal information received immediately after saccades that activates the mechanism for suppressing displacement detections. This implies that post-saccadic retinal information is an important factor with regard to visual stability. There are two major interpretations in relation to visual stability in the literature^21,22^. In the first interpretation, the visual system remaps the position information of the pre-saccadic target to the post-saccadic coordinates. Thanks to remapping, the pre- and post-target positions can be compared in a spatiotopic frame of reference, thus compensating the retinal shifts caused by the saccade. This is a model based on cancellation theory, stating that retinal displacements caused by saccades can be cancelled with extraretinal signals, such as a corollary discharge. In the second interpretation, the visual system assumes that the world is stable unless significant contradictory evidence is provided, such as the reappearance of a target in the blank condition in the present experiment^23^. This is called the theory of a stable world assumption. These two interpretations are not mutually exclusive, and both could contribute to visual stability. We discuss the relationship between these theories and our findings.

Wexler and Collins reported experimental results that can be explained by a combination of the cancellation theory and the stable world assumption theory^12^. They measured the threshold of displacement detection and compared it with the accuracy of saccades. Because the visual system cannot perform saccades perfectly due to, for example, extraocular muscle fatigue, there is an unpredictable gap between the saccadic target and the landing position (saccadic error) even after remapping to cancel the retinal displacements caused by saccades. The visual system has to judge whether this gap indicates an object movement or a saccadic error. Wexler and Collins proposed a model of how to make a judgment. If the post-saccadic target falls within a region of saccadic error, the visual system assumes that the target has not moved during the saccade (stable world assumption theory). If, on the other hand, the target position falls outside the saccadic error region, the visual system would judge that the target has moved and would discard the stable world assumption. The saccadic error region is an elliptical region, within which most saccades land, and the authors assumed that each person has a specific saccadic error region that is dependent on individual saccade accuracy. In this model, the visual system identifies the target movement based on remapping (cancellation theory) and the stable world assumption combined with the knowledge of the saccadic error region, and here we call it the *combined model*.

The results we obtained under the no-blank condition support the validity of the combined model. In the present experiment, the target displacement was fixed at 0.33°. The displacement was smaller than the average saccadic error of 2.56° (see Supplementary Table S1 online). This means that, under the no-blank condition, the post-saccadic target almost always fell within the region of saccadic errors. The detection rates under the no-blank condition were at the chance level, which is consistent with the prediction obtained with the combined model and, more specifically, the assumption of visual stability.

Our model extends the combined model and, moreover, proposes a specific mechanism to suppress displacement signals, causing SSD. Our results suggest that the suppression mechanism depends on the contrast of the post-saccadic stimulus. To explain these results, we assume that the signal for detecting displacement across saccades, conveyed via the M-pathway, is suppressed by the post-saccadic signal processed in the P-pathway. The model assumes remapping to allow the comparison of pre- and post-saccadic stimulus positions in spatial coordinates. In the combined model, the acceptance rates of the stable world assumption, which is experimentally measured as the detection rates of displacement, is expressed by the strength of the suppression. Thus, our model is a combined model but including a specific suppression mechanism.

**Figure 4:**
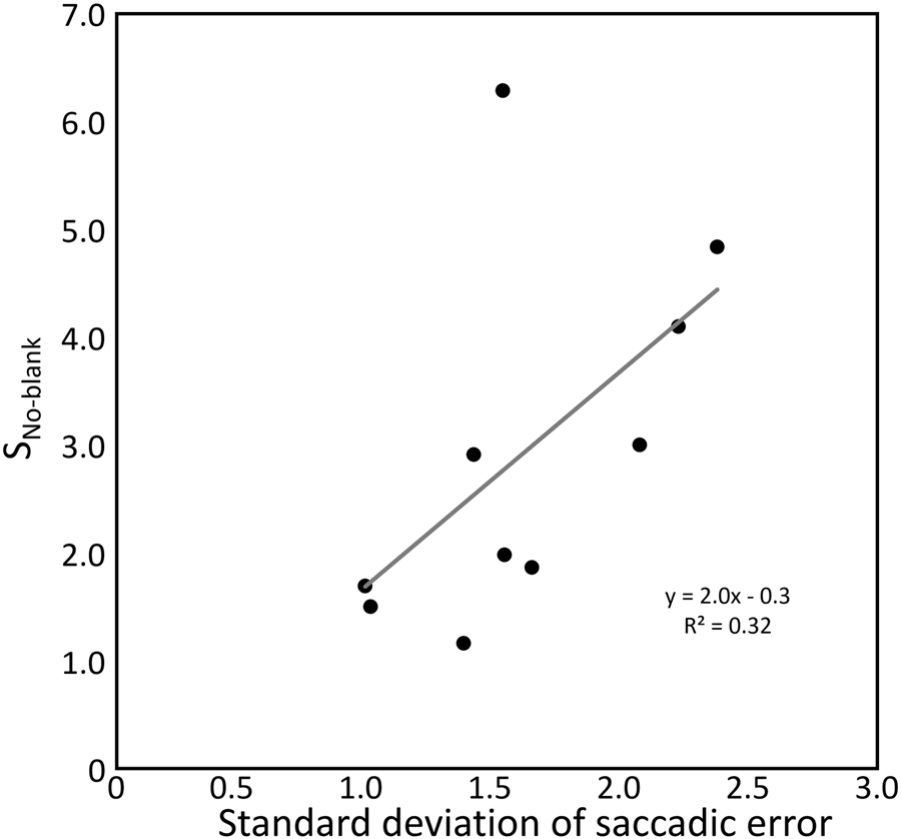
Relationship between standard deviation of saccadic error and strength of suppression. Saccadic error is defined as the distance from the mean location of the saccade goal to the location of the pre-saccadic target, and the strength of the saccadic suppression is defined by the value of S_No-blank_. To obtain the S_No-blank_, values, we fitted our model to the displacement detection sensitivity data of each observer. The parameters of our model were fixed to the values we obtained by fitting with average data over all observers, excluding S_No-blank_. Each dot represents observer’s data and the line represents the linear regression line. The correlation coefficient of the regression line is ρ= 0.56 (p = 0.045).

To examine whether saccadic error is related to the SSD as predicted by the combined model of Wexler and Collins, we analyzed the relationship between variation of saccadic error (standard deviation) and suppression weight (S_No-blank_) in our model. To obtain the values of S_No-blank_ of each observer, our model was fit to the displacement detection sensitivity data of the observer with the other parameters fixed to the values obtained for the average of all observer. Our analysis showed that observers with larger saccadic errors tend to have a higher suppression weight (S_No-blank_): the correlation coefficient between the two indexes is 0.56 (p=0.045) (Fig. 4). This supports Wexler and Collins’s model and also supports the suppression mechanism proposed in our model.

**Figure 5:**
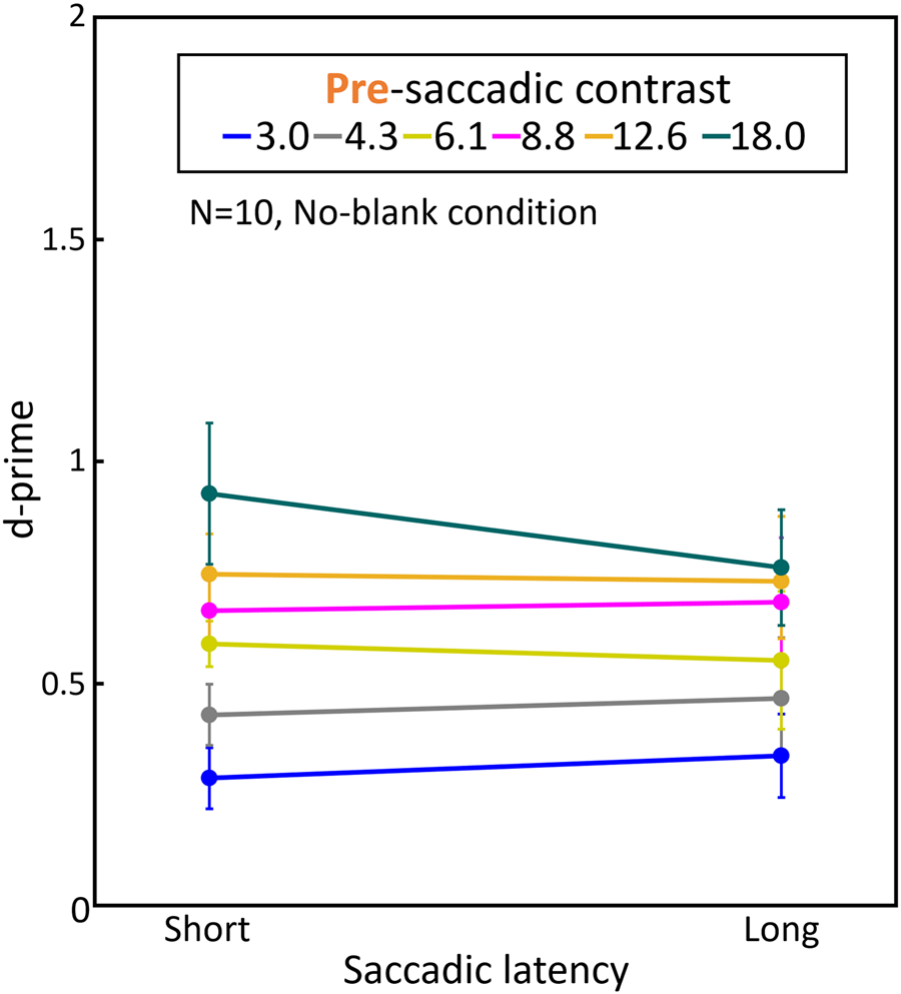
Influence of saccadic latency on the displacement detection performance for each pre-saccadic contrast condition. The response data for each trial under a no-blank condition were split in term of whether the saccadic latency was shorter or longer than the median value of each observer. The displacement detection performance for each pre-saccadic contrast is given as d’. Each line corresponds to different pre-saccadic contrast conditions. The error bars represent S.E.M.

Zimmermann and colleagues investigated the influence of the presentation time of the pre-saccadic target under low and high target contrast conditions. They demonstrated that increasing the presentation time improves the detection performance, while the target contrast did not influence the performance significantly but tended to improve it^24^, but see Matsumiya *et al*.^16^ and Collins^25^. To examine the influence of presentation time and contrast on detection performance for our data, we classified the response data based on the target presentation time. The presentation time varied with saccadic latency, and trials were classified into short and long saccadic latency presentation time groups separated at the median for each observer (see Supplementary Fig. S2 online). Figure 5 shows the displacement detection performance of the two groups for each pre-saccadic contrast under a no-blank condition. A two-way ANOVA analysis (6 pre-saccadic contrast x short/long saccade latency) revealed that there was a significant main effect of the pre-saccadic contrast (F(5,45) = 15.41, p < 0.001), but no significant main effect of the saccadic latency (F(1,9) = 0.33, p = 0.58) and no significant interaction between the pre-saccadic contrast and saccadic latency (F(5,45) = 1.22, p = 0.32). This result is inconsistent with that reported by Zimmermann *et al*. and this may suggest that different mechanisms are responsible for displacement detection in the two studies. Alternatively, different conclusions may be attributed to the effect of power of statistical test. The latency differences between short and long trials in the present experiment may be too small to find an effect, comparing with the original study (range between 100 to 1000 ms), and the number of observers in their experiment may not be large enough to show significant effect.

Although we assume SSD is caused by the suppression of M-pathway signals^15^, Castet *et al*. suggested that the reduction in the sensitivity for motion perception during a saccade can be explained by masking effects induced by retinal images before and/or after the saccade and that the M-pathway does not need to be suppressed^8,26^. Related to this suggestion, Zimmermann *et al*.^9^ demonstrated that the suppression of displacement detection across a saccade was similar to that of stimulus displacement disturbed by a random texture mask during fixation (see also Born^27^), suggesting that no specific suppression mechanism is necessary to explain SSD. Under such a condition, the displacement may have been detected not by the M-pathway signal, but by, for example, using information in the visual short-term memory^28^. Similarly, the displacement across saccade may be detected through the visual short-term memory. This is consistent with a phenomenon where changing a certain visual feature of the target before and after a saccade reduces the amplitude of SSD^29,30^, which cannot be explained by suppressing an early visual pathway, such as the M-pathway.

However, these findings and arguments do not necessarily relate to the suppression of the M-pathway or to other low-level motion/displacement detection mechanisms across the saccade. We assume a suppression mechanism for displacement detection around the saccade for the following reason. The blanking effect shows that there is a mechanism that detects displacement across the saccade with relatively good performance when a blank is interposed between pre- and post-saccadic targets. This corresponds to lengthening the interval of the mask or blank in a simulated saccade condition during fixation, which likely impairs, rather than improves, detection as motion perception studies have shown^31,32^. In addition, the assumption of the suppression of the motion signal around saccades appears to be unavoidable if we are to explain the lack of transient changes in visual perception with retinal changes caused by saccades. The lack of perceiving visual transients can never be achieved in simulated saccade conditions during fixation. It appears to be impossible to replace the target with a mask/blank (or a mask/blank with a target) without the perception of visual transients under simulated saccade conditions^4^. It also worth noting that masking effects during fixation are not always similar to the saccadic suppression. Duyck *et al*. showed a large masking effect in the simulated condition during fixation^33^ but also showed that the effect is not large enough to explain saccade-related suppression effects. Although the masking effect is one candidate for explaining SSD and there is no reason to deny its role, there is no direct evidence for denying a possible suppression mechanism at an early stage of visual processing based on the above discussion. Independent of whether a low or a high-level process is assumed, our result suggests the contribution of P-pathway related signals to the suppression of displacement perception across a saccade.

There is another type of interpretation, namely the assumption of a masking effect. The decremental effect of post-saccadic contrast can be explained with a backward masking by the post-saccade stimulus, affecting the representation of the pre-saccadic stimulus. If the post-saccadic target masks the remapped representation created by the pre-saccadic target, it is not possible to detect displacement. Such trans-saccadic and spatiotopic masking has been reported before^34–36^. Paeye *et al*. showed that a high contrast post-saccadic target produced effective masking (or overwriting) of the spatial representation of a pre-saccadic target after its remapping across a saccade^36^. In contrast, if the post-saccadic target contrast is sufficiently low, the pre-saccadic representation can survive after a saccade and the pre- and post-saccadic representations are fused. The present results may be explained by this interpretation as follows. With high post-saccadic contrasts, the pre-saccadic target representation is strongly masked, and the visual system cannot compare the pre- and post-saccadic target positions to detect target displacement. As the post-saccadic contrast decreases, the masking effect becomes weaker and the displacement can be detected more easily. This explanation is also consistent with our quantitative model while the blanking effect remains to be explained. If, in our model, there is suppression by the P-pathway of the position signal from the pre-saccadic target, the suppression can be recognized as trans-saccadic masking. In other words, our model assumes that trans-saccadic masking is due to P-pathway activity. Either a low- or a high-level process is concerned, our result suggests contribution of P-pathway related signals to suppression of displacement perception across saccade.

There is another related question of an assumption in our model. That is, whether there is a process with which to remap the spatial coordinate. Our model assumes the remapping process, which maintains the relationship between the retinal coordinates and the spatial coordinate in an appropriate manner. Due to the remapping, our motion detector can detect displacements across a saccade as in the same manner as a low-level motion process detects retinal displacements. The reason why we assume the remapping process is because the detection performance is recovered with a blank immediately after a saccade (blanking effect). The blanking effect is counter-intuitive and likely to relate to early visual processes rather than to late processes such as short-term memory, attention or feature tracking. Moreover, the success of predictions of behavioral results by our model suggests the existence of a remapping process, although the present results make no direct contribution to the question of remapping.

Our model only considers processing in the early visual pathways. However, since the information transmitted through the P-pathway is sent to the ventral pathway in the visual cortices, the model prediction may be related to the suppression effect influenced by the shape, surface feature and contrast polarity^29,30^ (while not by orientation^37)^, that is influence of visual features on trans-saccadic integration. It has also been demonstrated that a pre-saccadic stimulus influences the perception of visual features, such as the color, shape, or orientation, of a post-saccadic stimulus^38–42^. Furthermore, the blanking effect is known for the trans-saccadic discrimination of visual features. That is, discrimination tasks across saccades are improved by post-saccadic blanking^43–45^. Further investigation of the effects of other visual features on trans-saccadic perception including SSD and will reveal the mechanism of visual stability in more detail.

In summary, we discovered an impairment of displacement detection across a saccade by the post-saccadic stimulus. This finding suggests that the visual system actively suppresses displacement detection to achieve visual stability by using post-saccadic information. We demonstrated that the results can be explained by the suppression effect of P-pathway signals on M-pathway processing.

## Methods

### Observers

Eight male and two female observers ranging from 21 to 24 years old (mean age, 22 years), with normal or corrected to normal vision, participated in this experiment. They gave informed consent in accordance with the Code of Ethics of the World Medical Association (Declaration of Helsinki). Nine of them were naive to the purpose of this study. The other observer was one of the authors. This study was approved by the Ethics Committee of the Research Institute of Electrical Communication, Tohoku University.

### Apparatus

The observer’s head was fixed with a chin rest and the viewing distance was 45 cm. Visual stimuli were presented on a 21-inch CRT display (GDM-F520, Sony, refresh rate: 100 Hz). The observer’s eye position was measured with a limbus tracking device consisting of an infrared emitting diode and two photodiodes (T.K.K.2930a, Takei Scientific Instruments Co., Ltd.). The analog signal (voltage) from the device was digitized by ViSaGe and was sampled in synchronization with the monitor frame refresh timing (sampling rate: 100 Hz). To avoid time delays, we did not use digital and analog filters. Eye position was continuously measured during each trial and eye movement velocity was calculated online by a three-points-difference algorithm in which the velocity at time n was calculated from the eye position at time n-1 and time n + 1. The onset of a saccade was defined as the time at which the eye velocity exceeded 30°/s. Due to the velocity calculation method, saccade onset detection was always delayed by 1 frame (10 ms). In such saccade-triggered manipulations, there is a potential danger that pre-saccadic target is removed only after the end of the saccade. Because observers could then see the target displacement during fixation after saccade, this might lead to a seeming increase of detection sensitivity. To verify the timing in our experiment, we calculated the saccade offset time and target presented time. This analysis revealed that the removal of the pre-saccadic target (and, in the no-blank case, the appearance of the post-saccadic stimulus) was on average 32 ms before the saccade offset; only in 0.7% of trials the pre-saccadic target was extinguished after the saccade - these trials were excluded from further analysis. Also, trials were excluded if the saccade latency was shorter than 120 ms or longer than 400 ms. Standard deviations and averages of saccadic error were calculated for each observer and, if the saccadic error was longer or shorter than ±3 SD from the average, its trial was excluded. On the basis of these criterion, 6% of the trials were excluded from the analysis.

### Calibration of eye movement

Each session started with a calibration process. At the beginning of the calibration, five equally separated dots were presented sequentially along a horizontal line and a center dot was located at the center of the display. Observers were instructed to fixate on each point in turn and push a button when each fixation was completed. Horizontal eye positions were expressed as voltages when the button was pushed. The relationship between voltage and dot position was determined by a linear regression. If the regression coefficient was less than 0.9, the calibration procedure was repeated until the criterion was satisfied.

### Stimuli

The fixation point and the pre-saccadic and post-saccadic targets were disks 0.88° in diameter. The background luminance was constant at 21.4 cd/m^2^. Before the main experiment, we measured the contrast thresholds of the target detection in the central and peripheral visual fields for each observer (see Supplementary Fig. S1 online). The contrast here is Weber contrast, which is the ratio against the background, and so the contrast exceeded 100% in some cases. To take account of the difference in sensitivity between central and peripheral vision, the stimulus contrasts were determined by factors of threshold contrast. The contrast of the pre-saccadic target was 3, 4.3, 6.1, 8.8, 12.6, or 18 times the contrast threshold at peripheral vision and that of the post-saccadic target was 3, 4.3, 6.1, 8.8, 12.6, or 18 times the contrast threshold at central vision. The contrasts of the targets were randomly chosen for each trial, and measurements were performed for all the contrast combinations of the pre- and post-targets. The contrast of the fixation point was an average of the all of the pre-saccadic and post-saccadic target contrasts.

### Procedure

**Figure 6:**
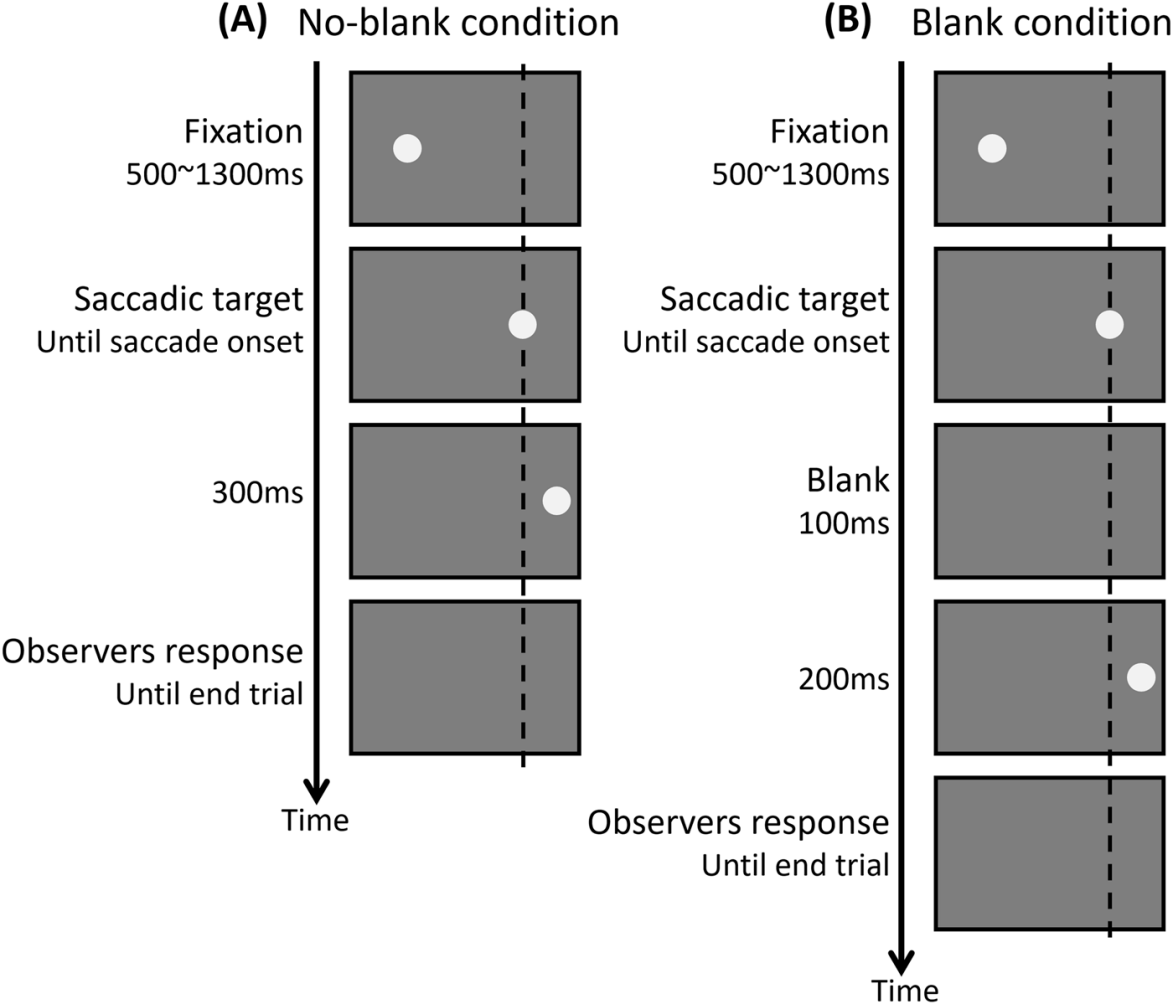
Experimental procedure. (**A**) No blank condition. Observers fixated at the fixation point and pressed a button to start the trials. After a random presentation interval (500–1300 ms), the fixation point disappeared, and the target was presented. Observers were instructed to make a saccade to the target. The saccadic target was removed with the saccade onset and immediately presented at a position 0.33° to the left or right of the original target position. The target stayed on screen until 300 ms after the displacement. (**B**) Blank condition. The procedure was the same as with the no blank condition except that a 100 ms blank was inserted between target offset and onset.

The experimental procedure is shown in Fig. 6. Initially, the fixation point was presented 8.9° to the left of the display center. The observer fixated on the fixation point and pressed a button to start the trial. The fixation point was disappeared after a randomly selected duration between 500 and 1300 ms, and the target was presented 8.9° to the right of the display center (17.8° to the right of the initial fixation point). The observer was instructed to make a saccade to the target as quickly as possible. The target was disappeared with the saccade onset. Under the no-blank condition, the target was displaced by 0.33° to the left or right after saccade onset and stayed until 300 ms after displacement. Under the blank condition, the target disappeared with saccade onset and reappeared 100 ms later, displaced by 0.33° to the left or right. The target was disappeared 200 ms after its reappearance. The observers pressed a button to report whether the target was displaced to the left or right of its original location. Each observer participated in twenty sessions, each included 144 trials (2 (blank or no-blank) × 6 (pre-saccadic contrast condition) × 6 (post-saccadic contrast condition) × 2 (left or right displacement)).

### Ethical statement

This study was approved by the Ethics Committee of the Research Institute of Electrical Communication, Tohoku University and conducted in accordance with the Code of Ethics of the World Medical Association (Declaration of Helsinki).

## Supplementary information

Supplementary Information.

## References

[1] Volkmann FC, Schick AM, Riggs LA (1968). Time course of visual inhibition during voluntary saccades. JOSA A..

[2] Riggs LA, Manning KA (1982). Saccadic suppression under conditions of whiteout. Invest Ophthalmol Vis. Sci..

[3] Bridgeman B, Hendry D, Stark L (1975). Failure to detect displacement of the visual world during saccadic eye movements. Vision Res..

[4] Shioiri S, Cavanagh P (1989). Saccadic suppression of low-level motion. Vision Res..

[5] Uchikawa K, Sato M (1995). Saccadic suppression of achromatic and chromatic responses measured by increment-threshold spectral sensitivity. JOSA A..

[6] Campbell FW, Wurtz RH (1978). Saccadic omission: why we do not see a grey-out during a saccadic eye movement. Vision Res.

[7] Wurtz RH (2008). Neuronal mechanisms of visual stability. Vision Res.

[8] Castet E, Jeanjean S, Masson GS (2002). Motion perception of saccade-induced retinal translation. Proc Natl Acad Sci USA.

[9] Zimmermann E, Born S, Fink GR, Cavanagh P (2014). Masking produces compression of space and time in the absence of eye movements. J Neurophysiol.

[10] Grüsser O-J, Krizc A, Weiss L-R (1987). Afterimage movement during saccades in the dark. Vision Res..

[11] Bridgeman B, Macknik SL (1995). Saccadic suppression relies on luminance information. Psychol Res..

[12] Wexler M, Collins T (2014). Orthogonal steps relieve saccadic suppression. J Vision..

[13] Niemeier M, Crawford JD, Tweed DB (2003). Optimal transsaccadic integration explains distorted spatial perception. Nature.

[14] Deubel H, Schneider WX, Bridgeman B (1996). Postsaccadic target blanking prevents saccadic suppression of image displacement. Vision Res..

[15] Burr DC, Morrone MC, Ross J (1994). Selective suppression of the magnocellular visual pathway during saccadic eye movements. Nature.

[16] Matsumiya K, Sato M, Shioiri S (2016). Contrast dependence of saccadic blanking and landmark effects. Vision Res.

[17] Sclar G, Maunsell JHR, Lennie P (1990). Coding of image contrast in central visual pathways of the macaque monkey. Vision Res..

[18] Naka KI, Rushton WAH (1966). S-potentials from colour units in the retina of fish (Cyprinidae). J Physiol..

[19] Shioiri S, Ito S, Sakurai K, Yaguchi H (2002). Detection of relative and uniform motion. J Opt Soc Am..

[20] Reichardt WE, Schlögl RW (1988). A two dimensional field theory for motion computation. Biol. Cybern..

[21] Sperry RW (1950). Neural basis of the spontaneous optokinetic response produced by visual inversion. J Comp Physiol..

[22] Von Holst E, Mittelstaedt H (1950). Das reafferenzprincip. Naturwissenschaften..

[23] MacKay DM (1972). Voluntary eye movements as questions. Bibl Ophthalmol..

[24] Zimmermann E, Morrone MC, Burr DC (2013). Spatial position information accumulates steadily over time. J. Neurosci..

[25] Collins T (2016). The spatiotopic representation of visual objects across time. Atten Percept Psychophys.

[26] Castet E, Masson GS (2000). Motion perception during saccadic eye movements. Nat Neurosci.

[27] Born, S. Saccadic Suppression of Displacement Does Not Reflect a Saccade-Specific Bias to Assume Stability. *Vision (Basel)* **3**, 10.3390/vision3040049 (2019). 10.3390/vision3040049 PMC6969937 31735850

[28] Hollingworth A, Richard AM, Luck SJ (2008). Understanding the function of visual short-term memory: transsaccadic memory, object correspondence, and gaze correction. J Exp Psychol Gen.

[29] Demeyer M, De Graef P, Wagemans J, Verfaillie K (2010). Object form discontinuity facilitates displacement discrimination across saccades. J. Vision..

[30] Tas AC, Moore C, Hollingworth A (2012). An object-mediated updating account of insensitivity to. J Vis..

[31] Braddick O (1974). A short-range process in apparent motion. Vision Res.

[32] Shioiri S, Cavanagh P (1990). ISI produces reverse apparent motion. Vision Res.

[33] Duyck M, Wexler M, Castet E, Collins T (2018). Motion Masking by Stationary Objects: A Study of Simulated Saccades. Iperception.

[34] De Pisapia N, Kaunitz L, Melcher D (2010). Backward masking and unmasking across saccadic eye movements. Curr. Biol..

[35] Germeys F, De Graef P, Van Eccelpoel C, Verfaillie K (2010). The visual analog: Evidence for a preattentive representation across saccades. J.Vision..

[36] Paeye C, Collins T, Cavanagh P (2017). Transsaccadic perceptual fusion. J Vision..

[37] Balp R, Waszak F, Collins T (2019). Remapping versus short-term memory in visual stability across saccades. Atten Percept Psychophys.

[38] Poth, C. H., Herwig, A. & Schneider, W. X. Breaking Object Correspondence Across Saccadic Eye Movements Deteriorates Object Recognition. *Front Syst Neurosci* **9** (2015). 10.3389/fnsys.2015.00176 PMC4685059 26732235

[39] Poth CH, Schneider WX (2016). Breaking object correspondence across saccades impairs object recognition: The role of color and luminance. j Vis..

[40] Oostwoud Wijdenes L, Marshall L, Bays PM (2015). Evidence for Optimal Integration of Visual Feature Representations across Saccades. J Neuroschi..

[41] Demeyer M, De Graef P, Wagemans J, Verfaillie K (2010). Parametric integration of visual form across saccades. Vision Res..

[42] Fornaciai M, Binda P, Cicchini GM (2018). Trans-saccadic integration of orientation information. J Vis..

[43] Tas AC, Moore CM, Hollingworth A (2014). The representation of the saccade target object depends on visual stability. Vis cogn..

[44] Weiß K, Schneider WX, Herwig A (2015). A “blanking effect” for surface features: Transsaccadic spatial-frequency discrimination is improved by postsaccadic blanking. Atten Percept Psychophys..

[45] Grzeczkowski, L., Deubel, H. & Szinte, M. Stimulus blanking reveals transsaccadic feature transfer. 10.1101/819110 (2019). 10.1038/s41598-020-75717-y PMC7596086 33122762

