# Supplementary material for: Displacement detection is suppressed by the post-saccadic stimulus
Source: Sci Rep. 2020 Jun 9;10:9273. doi: 10.1038/s41598-020-66216-1 (PMC7283269; doi:10.1038/s41598-020-66216-1)
Supplement: Supplementary file 1 — Supplementary Information. [file 41598_2020_66216_MOESM1_ESM.pdf]

Displacement detection is suppressed by the post-saccadic stimulus

Shuhei Takano[1, 2] (stakano@riec.tohoku.ac.jp)

Kazumichi Matsumiya[1, 2] (matsumiya@cp.is.tohoku.ac.jp)

Chia-huei Tseng[1, 2] (tseng@riec.tohoku.ac.jp)

Ichiro Kuriki[1, 2] (ikuriki@riec.tohoku.ac.jp)

Heiner Deubel[3] (heiner.deubel@psy.lmu.de)

Satoshi Shioiri[1, 2, *] (shioiri@riec.tohoku.ac.jp)

1. Graduate School of Information Sciences, Tohoku University, 6-3-09 Aramaki aza Aoba, Aoba-ku Sendai 980-8579, Japan
2. Research Institute of Electrical Communication, Tohoku University, 2-1-1 Katahira, Aoba-ku, Sendai 980-8577, Japan
3. Department Psychologie, Ludwig-Maximilians-Universität, Leopoldstr, 13 D-80802 München, Germany

***Corresponding Author:**

Satoshi Shioiri

Research Institute of Electrical Communication,

Tohoku University,

2-1-1 Katahira, Aoba-ku, Sendai 980-8577, Japan

TEL.+81-22-217-5468 / FAX.+81-22-217-5471

shioiri@riec.tohoku.ac.jp

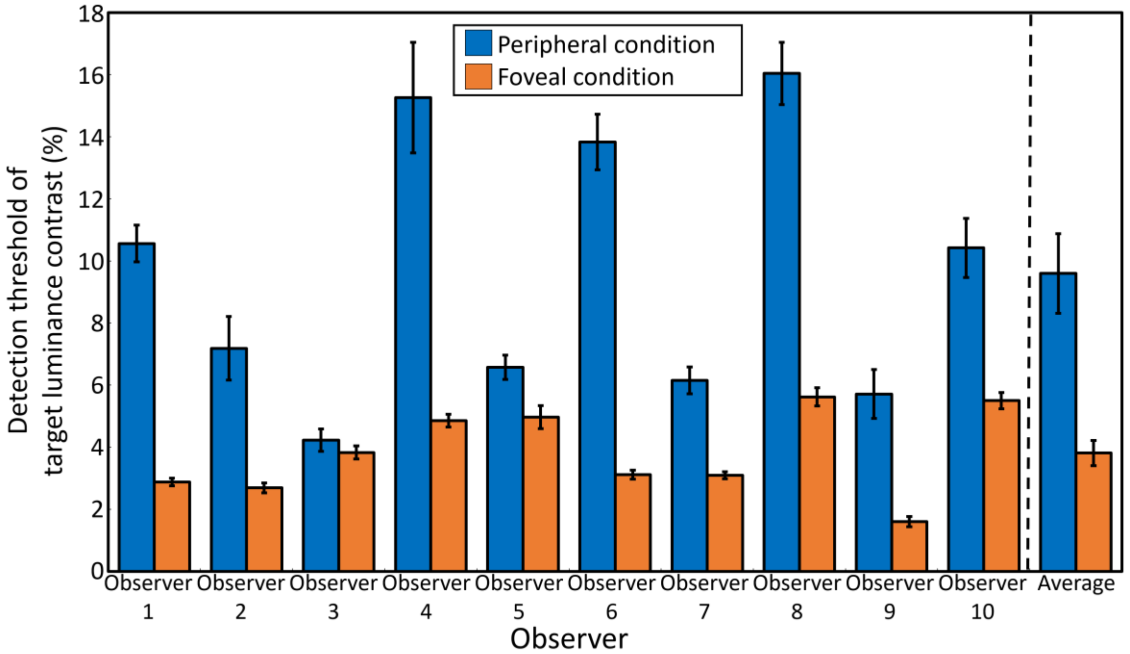

Figure S1. Detection threshold of luminance contrast for each observers and grand average value. Observers adjusted the luminance contrast of the target to the lowest value at which they could detect the target. In the peripheral vision condition, observers fixated a dot which was presented at horizontally 8.9° away from center of the monitor and adjusted contrast of a target contrast which was presented horizontally 17.8° away from the fixation point. In the central vision condition, observers gazed the target directly and adjusted its contrast. Observers conducted 15 trials each in peripheral and foveal conditions, and the detection thresholds were defined as the average of these values. The blue bars represent the detection threshold in peripheral vision (average threshold: 9.6%) and the orange bars represent the detection threshold in central vision (average threshold: 3.8%). The error bars for each observer represent S.E.M. of 15 trials. The error bar of the average value represents S.E.M. among observers.

Observer	1	2	3	4	5	6	7	8	9	10	AVE
Error (deg)	1.27	2.57	4.07	3.75	2.15	3.45	2.04	2.59	2.40	1.27	2.56

Table S1. The average value of the saccadic error (visual angle) over the all trials. The saccadic error is defined as the distance from the mean location of the saccade goal to the location of the pre-saccadic target. Error in this table indicates the average saccadic error across all trials for each observer. The grand average across all observers is also provided (AVE=2.56deg). All observers made larger saccadic errors than the target displacement (0.33°) across the saccades in this experiment.

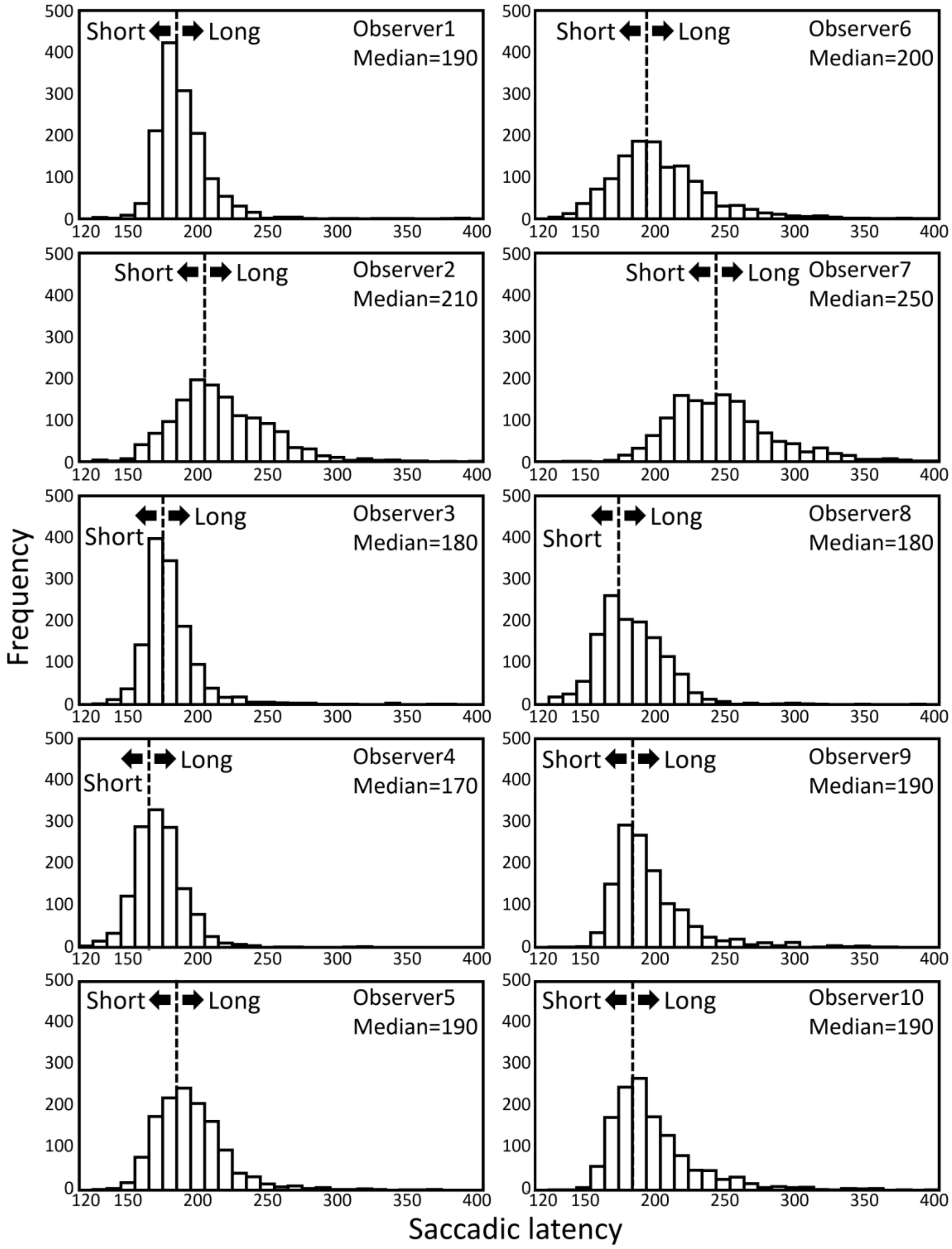

Figure S2. Distribution of saccadic latencies under the no-blank condition. Saccadic latency is defined as the time from pre-saccadic target presentation to saccade onset. Histograms and median values are calculated for individual observers.
